# A Novel Approach of Combining Methylene Blue Photodynamic Inactivation, Photobiomodulation and Oral Ingested Methylene Blue in COVID-19 Management: A Pilot Clinical Study with 12-Month Follow-Up

**DOI:** 10.3390/antiox11112211

**Published:** 2022-11-08

**Authors:** Juliette Hepburn, Susan Williams-Lockhart, René Jean Bensadoun, Reem Hanna

**Affiliations:** 1 Luminnova Health, 34 Harbour Bay Plaza, East Bay Street, Nassau P.O. Box N-1081, Bahamas; 2 Chief Medical Officer, Capriata Health and Wellness, Nassau P.O. Box N-9792, Bahamas; 3 Centre De Haute Energie, Department of Oncology Radiology, 10 Boulevard Pasteur, 06000 Nice, France; 4 Department of Surgical Sciences and Integrated Diagnostics, Laser Therapy Centre, University of Genoa, Vaile Benedetto XV, 6, 16132 Genoa, Italy; 5 Department of Restorative Dental Sciences, UCL-Eastman Dental Institute, Faculty of Medical Sciences, Rockefeller Building, London WC1E 6DE, UK; 6 Department of Oral Surgery, King’s College Hospital NHS Foundation Trust, Denmark Hill, London SE5 9RS, UK

**Keywords:** COVID-19, photodynamic inactivation, photooxidants, photobiomodulation, PCR, oral methylene blue, viral load, oxidative stress, SARS CoV-2 genes expression, light emitted diodes

## Abstract

Coronavirus disease 2019 (COVID-19) caused by SARS-CoV-2 virus was first recognized in late 2019 and remains a significant threat. We therefore assessed the use of local methylene blue photodynamic viral inactivation (MB-PDI) in the oral and nasal cavities, in combination with the systemic anti-viral, anti-inflammatory and antioxidant actions of orally ingested methylene blue (MB) and photobiomodulation (PBM) for COVID-19 disease. The proposed protocol leverages the separate and combined effects of MB and 660nm red light emitted diode (LED) to comprehensively address the pathophysiological sequelae of COVID-19. A total of eight pilot subjects with COVID-19 disease were treated in the Bahamas over the period June 2021–August 2021, using a remote care program that was developed for this purpose. Although not a pre-requisite for inclusion, none of the subjects had received any COVID-19 vaccination prior to commencing the study. Clinical outcome assessment tools included serial cycle threshold measurements as a surrogate estimate of viral load; serial online questionnaires to document symptom response and adverse effects; and a one-year follow-up survey to assess long-term outcomes. All subjects received MB-PDI to target the main sites of viral entry in the nose and mouth. This was the central component of the treatment protocol with the addition of orally ingested MB and/or PBM based on clinical requirements. The mucosal surfaces were irradiated with 660 nm LED in a continuous emission mode at energy density of 49 J/cm^2^ for PDI and 4.9 J/cm^2^ for PBM. Although our pilot subjects had significant co-morbidities, extremely high viral loads and moderately severe symptoms during the Delta phase of the pandemic, the response to treatment was highly encouraging. Rapid reductions in viral loads were observed and negative PCR tests were documented within a median of 4 days. These laboratory findings occurred in parallel with significant clinical improvement, mostly within 12–24 h of commencing the treatment protocol. There were no significant adverse effects and none of the subjects who completed the protocol required in-patient hospitalization. The outcomes were similarly encouraging at one-year follow-up with virtual absence of “long COVID” symptoms or of COVID-19 re-infection. Our results indicate that the protocols may be a safe and promising approach to challenging COVID-19 disease. Moreover, due its broad spectrum of activity, this approach has the potential to address the prevailing and future COVID-19 variants and other infections transmitted via the upper respiratory tract. Extensive studies with a large cohort are warranted to validate our results.

## 1. Introduction

More than two years after declaration of a pandemic, COVID-19 remains a serious concern globally, with successive waves of increasingly more resistant variants [1]. There is an urgent need for effective anti-viral therapy which is not variant-specific and can therefore remain effective despite the rapid emergence of variants of concern.

Photodynamic therapy offers great potential in this regard with established ability to address bacterial, fungal, protozoan and viral infections, with very low likelihood of drug resistance due to its rapid, multi-targeted cidal effects [2].

A fundamental aspect of COVID-19 disease is the tissue and organ damage caused by a hyper-inflammatory state [3] often referred to as a cytokine storm. Oral and inhaled steroids have been widely used to address this issue but using broad immunosuppressive agents in the context of severe infection may have potential drawbacks. Methylene blue (MB) and photobiomodulation both offer anti-inflammatory and immune modulating effects which appear to be additive and of significant benefit in COVID-19 disease [3,4]. In fact, MB appears to ameliorate COVID-19 disease at virtually every stage of the pathophysiological process as documented by Dabholkar et al., 2021 [5] and outlined in Table 1.

### 1.1. Oral Methylene Blue (MB)

MB is FDA approved as a lifesaving treatment for methemoglobinemia. There is growing evidence of many other health benefits, particularly as an enhancer of mitochondrial function, cognitive function and mental health [6,7,8,9]. MB also exerts an impressive range of beneficial actions in COVID-19 disease (Table 1) including anti-viral [10,11], anti-inflammatory [12,13] and anti-thrombotic [14] activity. As such, the use of a single drug MB appears to fulfill the cornerstones of early intervention in COVID-19 disease, which is otherwise fulfilled by sequential, multi-drug treatment [15].

With respect to anti-viral activity, MB has been reported to inhibit the attachment of the SARS-CoV-2 spike protein to the angiotensin-converting enzyme-2 (ACE2) receptor even at very low, physiologically relevant concentrations (IC_50_ = 3 μM) [16]. MB increases endosomal and lysosomal pH and reduces the viral entry and uncoating further [17,18]. MB is a zinc ionophore and promotes the diffusion of zinc across the viral envelope. This may inhibit viral replication by blocking RNA dependent RNA polymerase [19]. The anti-inflammatory effects of MB appear to be mediated in part by inhibition of macrophage NLRP3 inflammasome complex, which has a profound effect in reducing a large number of inflammatory cytokines [20,21,22]. Other anti-inflammatory actions include reduction in bradykinin activity by reducing excess nitric oxide (NO). MB is also a highly effective scavenger of highly reactive pro-inflammatory oxygen and nitrogen species [5].

The formation of micro-clots is a critically important sequela in the pathogenesis of COVID-19 disease and is responsible for a number of severe outcomes and death. MB is a potential inhibitor of platelet aggregation especially in the context of hyper-inflammation and may therefore be of significant benefit in this regard [14].

MB has proven to possess intrinsic anti-bacterial [23], anti-fungal [24] and anti-viral activity [25]. This may be further enhanced by photo-activation of orally ingested MB within the bloodstream. A reduction in secondary infection by MB may significantly reduce disease burden and mortality due to COVID-19 [26].

Based on the actions described above it is not surprising that improvement of fever, malaise and hypoxia was often observed within a few hours (h) of oral administration of MB in study subjects. Furthermore, MB is generally well-tolerated and regarded as a very safe drug. The two main precautions are a limited number of drug interactions and G6PD deficiency. The major concern relating to MB drug interactions is serotonin syndrome. This appears to be a significant factor associated with intravenous infusion [27], as opposed to oral administration [28]. In fact, the FDA updated its recommendations in this regard several months after its initial warning, but this is not widely appreciated [29]. With respect to the risk in G6PD deficiency, administration of MB based on short courses of treatment appear to be well tolerated in adults and children, including those with G6PD deficiency and at significantly higher doses than those utilized in our study [30].

### 1.2. Photobiomodulation (PBM)

A recent evidence-based review conducted by Hanna et al., 2021 highlighted the molecular mechanisms of COVID-19 and potential therapeutic strategies of which phototherapy can be considered, as a potential treatment modality in COVID-19 management [3,31].

The literature has well-documented that photobiomodulation (PBM) therapy acts as an immunomodulator, inducing antioxidant and anti-inflammatory effects [32,33,34]. It is effective in reducing inflammatory cytokines through PBM cellular signaling and Ca^2+^ sensitivity, which accounts for its anti-inflammatory effects [32,34]. Additionally, studies have shown PBM effectiveness in alleviating pain [35,36,37], reducing oxidative stress and accelerating wound healing [38,39,40].

**Figure 1:**
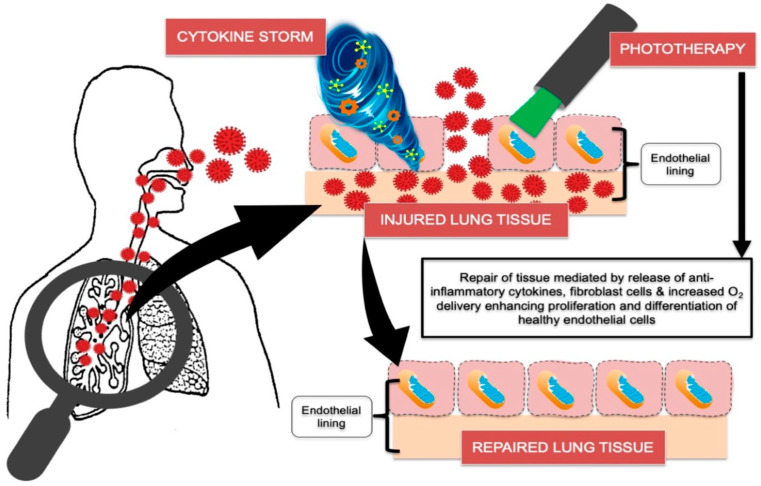
Shows COVID-19 mechanism of action and the role of phototherapy in lung tissue repair. Adapted with permission of Hanna et al., 2020 [3].

The review conducted by Hanna et al. 2020 [3] documented the molecular and cellular activities of phototherapy in regulating COVID-19 induced cytokine storm (Figure 1). The COVID-19 virus can dysregulate the immune response resulting in severe disease associated with intensive care unit (ICU) admission. This is related to excessive levels of IL-1β,4,6 and10, IFN-γ and TNF-α, which correlate to Th1, Th-2 and innate immune responses, respectively. Interestingly, several studies have shown PBM effectiveness in modulating the pulmonary immune responses in management of chronic obstructive pulmonary diseases (COPD) [34,41].

Additionally, the actions of both MB and PBM on mitochondrial function are widely recognized. Several studies have shown the impact of dysfunctional mitochondria on the immune response. Recent studies revealed that human alveolar epithelial cells with dysfunctional mitochondria displayed increased production of pro-inflammatory cytokines (CXCL-8, IL-6, CCL20, CCL3, CCL4 and IL-12) all of which were found to be increased in COVID-19 [42,43].

In contrast to systemic steroids which broadly suppress the immune response, PBM stimulates complexes of the mitochondrial respiratory chain to optimize and modulate the immune response, resulting in an improvement of its ability to eradicate infection via stimulation of the innate and adaptive immune cells [44,45,46], while providing an inhibitory effect on excessive inflammation [3]. Moreover, PBM can prompt cellular regeneration in every organ [47,48,49,50,51]. Thus, the use of PBM offers significant advantages over systemic steroids.

PBM is often applied to local target tissues to exploit its many beneficial effects. However, several reports indicate that a multi-organ systemic effect of PBM can be achieved by directly targeting the blood circulation. These efforts were pioneered by Russian scientists in 1981 and other scientists have now documented cardiovascular and other organ system benefits [52,53,54,55], including optimized immunological response [56,57,58], increased erythrocyte deformability [59], improved endothelial function [60,61] and reduced thrombocyte aggregation [62] with improved tissue oxygenation [63,64,65,66]. All of these multi-system PBM effects may be highly beneficial in the context of COVID-19 disease [61,67].

Sublingual and nasal PBM provide a non-invasive alternative means of targeting the blood circulation and have been shown to provide comparable effects to intravenous blood irradiation [68,69]. Absorption of the light energy is significantly enhanced by the rich vascular circulation and reduced quantity of melanin at these sites in contrast to a transdermal route of administering the light energy density. The sublingual route is particularly well suited due to the size and number of blood vessels at this site, the very thin overlying epithelium and absence of hair follicles. While it is possible to achieve PBM of circulating blood transcutaneously, this requires exposure of large areas of the skin and/or longer exposure times. This latter approach is not readily available in a home setting to treat a highly infectious disease.

### 1.3. Local Photodynamic Viral Inactivation

Notwithstanding the multiple additive benefits of MB and PBM, the authors consider photodynamic inactivation (PDI) to be the central and critically important component of our treatment protocol. It is noteworthy that PDI has a very long history of use in various clinical applications and is regarded as a very safe method of treatment including on mucosal surfaces [70,71,72].

PDI involves activation of a photosensitizer compound by a compatible light energy in the presence of oxygen [73,74]. The most appropriate photosensitizer (PS) for PDI in the respiratory tract seems to be the phenothiazine derivative MB based on its good performance and safety in various clinical therapeutic uses. It was therefore utilized in our study. MB has a range of absorption within the visible light spectrum between λ 609–690 nm with a peak absorption curve from λ 632 to λ 664 nm [75].

MB-PDI results in oxidation of carbonyl moieties on proteins; oxidation of guanine residues; and single strand breakage and cross-linkage of RNA [76,77,78]. The broad based and rapid damage to viral structures may account for the lack of elicited microbial resistance which is a critically important advantage of the use of PDI as an anti-microbial measure [79,80]. This is in contrast with single target anti-viral measures which are far more susceptible to microbial resistance. Furthermore, as SARS-CoV-2 is an enveloped virus, it is particularly sensitive to PDI [81].

A systematic review by Dalvi et al., 2021 of several in vitro, in vivo and clinical studies has confirmed the significant efficacy of MB-PDI, as an antimicrobial measure [71]. MB-PDI has been successfully employed to inactivate pathogens in blood plasma [82,83]. Based on this action, the Theraflex-MB photodynamic system has been deployed as a commercial pathogen decontamination solution by blood banks. A high level of efficacy of MB PDI against the respiratory West Nile virus has also been demonstrated in vitro [84] and in vivo [85]. Papin et al., 2005 [85] confirmed a significant increase in the survival of mice infected with West Nile virus when treated with MB-PDI. This effect was equally observed in immune-competent and severe-combined immune deficient mice and may have important implications for targeting SARS-CoV-2 infection in immunocompromised patients.

Moreover, as the SARS-CoV-2 infection and many other respiratory viral infections appear to involve predominantly upper airway disease at the early stages of infection, targeting effective treatment to these mucosal surfaces appears logical. The application of local anti-microbial PDI to the oro-pharynx and nasal passages for SARS-CoV-2 disease, is supported by evidence that ACE-2 positive epithelial cell lining salivary gland ducts are early target cells of SARS-CoV-1 and SARS-CoV-2, and a likely source of the viral particles detected in saliva droplets, particularly during the early stages of infection [86,87].

A clinical case series conducted by Woelfel et al., 2020 showed that pharyngeal shedding of SARS-CoV-2 was very high during the first week of symptoms with a peak at 7.11 × 10^8^ RNA copies per throat swab on day four. Within one week of symptom onset, a significant number of viruses were bound to ACE2 receptors in the mucosa of the oral cavity and both throat and nasal cavities [88]. Schikora et al., 2020 [89] proposed that as long as viruses are localized at these sites, they are easily accessible to locally administered PDI. As a consequence, the number of viruses, which can potentially seed to the lower respiratory tract and other organs can be reduced with reduced systemic viral load and improved clinical outcomes [90]. Therefore, local PDI of COVID-19 at this stage of infection may offer substantial benefit. Furthermore, by reducing the viral load in the mouth and nose to undetectable levels, the patient may also be significantly less likely to transmit the virus to others [91].

Another potential benefit of local PDT is broad based viral antigen presentation and possible promotion of mucosal and systemic immunity. This phenomenon has been described in vivo in the context of anti-tumor therapy [92] and bacterial infection [93] whereby PDT induced a highly potent antigen specific immune response capable of inducing memory immunity. It may also play a role in the context of COVID-19 infection. A study by Schikora et al. (2020) documented that SARS-CoV antibodies response developed in 96% of actively treated subjects four weeks after a single oral/nasal MB-PDT treatment [89]. We should note that antibody tests available in Germany at the time of the study were not able to distinguish antibody responses between SARS-CoV-1 and SARS-CoV-2, but it was assumed that the antibody response was related to SARS-CoV-2 infection.

### 1.4. Pilot Study Aims and Objectives

This pilot study was modelled on two preceding successful clinical studies which employed PDI in the early stages of COVID-19 infection [89,94]. The aim was to confirm the benefits of this approach in the local population in the Bahamas. In view of the highly infectious nature of COVID-19 infection, a home-based LED device was designed by one of the authors for treatment of COVID-19 and other infections initiated via the upper respiratory tract. This approach was chosen to improve healthcare team safety and for patient convenience. The present pilot study was conducted to evaluate the feasibility of this home-based device to administer MB-PDI in combination with PBM and orally ingested MB in the early stages of COVID-19 infection, aiming to reduce COVID-19 viral load, symptoms and expedite recovery. A one-year follow-up period was included to assess the long-term response to treatment in addition to the immediate outcomes. The objectives of our study were as follows:

1. To confirm the reduction in viral load with the use of serial quantitative polymerase chain reaction (PCR) tests (cycle threshold (CT) measurements).
2. To evaluate symptom progression and recovery time over follow up time points.
3. To appraise the feasibility and convenience of the home-based device and treatment of COVID-19 disease.
4. To assess the long-term outcomes following treatment.

## 2. Materials and Methods

### 2.1. Pilot Study Design and Ethical Approval

A total of eight subjects with COVID-19 infection were tested and treated for COVID-19 disease over the period June 2021–August 2021.

The study was conducted in accordance with the Declaration of Helsinki and based on the initial results of the exploratory cases described, approved was granted by the National COVID-19 Medical Ethics Committee in the Bahamas on 18 July 2021 to conduct a standardized case series study (Research Identification Number MROS/311971/PC).

#### 2.1.1. Eligibility Criteria

##### Inclusion Criteria

1. Subjects who were residents of Nassau, Bahamas, presenting with COVID-19 symptoms of 10 days or less with a positive test for COVID-19, using reverse transcription polymerase chain reaction (RT-PCR) test.
2. Subjects were at least 18 years of age, of both genders, and of any ethnicity.
3. Subjects with or without systemic diseases (ASA I and II).

##### Exclusion Criteria

1. Concomitant therapy: patients who were taking any FDA/MHRA/EMA/WHO approved anti-viral drugs. (Note: as Ivermectin, hydroxychloroquine and N-acetyl cysteine were not formally approved or recognized COVID-19 treatments at the time this pilot study was conducted, patients were not excluded if they were already taking these medications/supplements. However, it was clearly annotated if any of these medications/supplements were administered, thereby a response with and without these agents could be assessed).
2. Any enrolled subjects who needed urgent medical care, such as O_2_ saturation < 90% or severe hypertension (i.e., subjects medically compromised).
3. Subjects who were unable to properly comply with the treatment instructions and complete the course of the treatment protocols.

### 2.2. Assessment Tools of Clinical Outcomes

#### 2.2.1. Cycle Threshold Tests

The measurements of the PCR-CT were obtained from a single lab for all tests carried out. All the samples were maintained between 2 and 8 degrees Celsius and delivered to the laboratory within 12 h or less. All samples were processed by the same professional laboratory using the Bioneer AccuPower SARS-CoV-2 Multiplex Real Time RT-PCR Kit manufactured by Bioneer Corporation, Daejeon, South Korea. The following two gene target groups were measured: the E gene and SARS-CoV-2 genes (RdRp and/or N gene). The E gene negative threshold value was 35 and SARS-CoV-2 gene negative threshold value was 34. Estimated changes in the viral load were calculated using the initial SARS-CoV-2 gene target CT and the negative SARS-CoV-2 gene target threshold (34). This calculation is based a log base 2 scale: The estimated viral load reduction (EVLR) was calculated with the following formula.

EVLR = 2 ^(negative threshold CT value − initial CT value)^

We recognize that CT values are not a direct measurement of viral load present but can provide a useful measure of the relative viral loads over time in a given patient, particularly if a standardized swab technique is utilized and specimens are processed by the same laboratory professional using the same test equipment.

#### 2.2.2. Patient Clinical Data

Patient clinical data were obtained remotely by serial phone and serial online questionnaires. Online questionnaires confirmed COVID-19 vaccination history, past medical history, medications, supplements, allergies, symptom onset, graded symptom severity and the presence of any other symptomatic persons in the household. Follow up questionnaires were requested 12-hourly for the first 24 h and then daily until recovery time. Each follow-up questionnaire specifically elicited any adverse effects or difficulties experienced with the home treatments. The follow up questionnaire also graded symptom severity and provided a free text section for any additional symptoms or comments regarding clinical progress. Home visits were organized in response to any concerning signals indicated during the telephone review calls or from the online questionnaire feedback.

All of the above data were collected and analyzed by an independent research assistant who was not involved in either the study design or in subjects’ treatments. All the subjects were given a number for identification. A single experienced clinician was involved in treatment and in overseeing the subjects’ progress.

#### 2.2.3. Home Delivery of Treatment Supplies

The treatment supplies were delivered to subjects’ homes including the treatment device, MB solution and oral MB capsules, if indicated.

#### 2.2.4. Description of the Treatment Device

**Figure 2:**
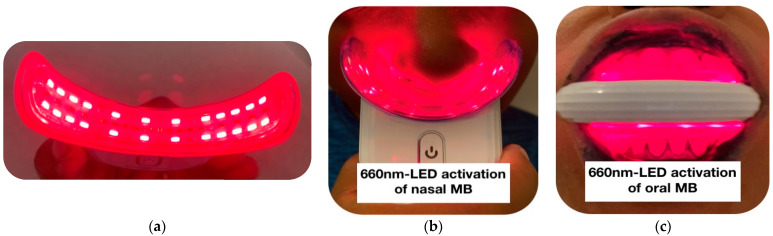
Shows light emitted diode (LED) home-device that was utilized in the study. Photo (**a**): shows the device illumined with 24 LEDs. Photos (**b**,**c**) illustrate the device application underneath the nostrils and inside the oral cavity, respectively, and were illuminated after methylene blue (MB) solution was topically administered in both cavities.

Our pilot study utilized a treatment device that is shown in Figure 2, in contrast to the laser device that was utilized in German MB-PDI clinical trial [89]. We utilized λ 660 nm LED light to stimulate MB solution administered in both the nasal and oral cavities. The advantages of the utilized LED device were related to cost, convenience, eye-safety and reduced the risk of cross-infection. However, one drawback of the LED light source is the attenuation in energy density observed over even very short device-target distances. This poses a greater challenge within the oro-pharynx.

The pilot study device provided a non-thermal, non-coherent light source emitting non-ionizing radiation within the visible light spectrum. The device parameters are as follows: wavelength: λ 660 nm, total power output: 2928 mW, irradiance 163 mW/cm^2^. The duration of each treatment session to both the nasal and oro-pharynx was 5 min (300 s) resulting in a maximum calculated energy density of 49 J/cm^2^ emitted by the device. Effective energy density lessened with increasing distance from the device, particularly at the midline of the tongue and throat. However, the fact that subjects had to elevate the tongue (which heavily adsorbed the MB solution), in order to avoid discomfort during the PDI treatment session, indicates that an adequate light energy dose reached this area. Of note, the MB solution and the light energy alone did not have this effect.

Schikora et al., 2020 [89] reported that the energy density administered to the oro-pharynx was 360 J/cm^2^ over 25 min using a laser probe with an aperture of approximately 1 cm^2^. In the present study the laser probe was held just outside the subject’s mouth which was held wide open for the duration of the treatment. As a result, the emitted energy was dispersed over a wider area. The average surface area of the adult mouth is reported to be approximately 215 cm^2^, but this is likely to be a significant overestimate in the context of light irradiation, as the tongue papillae account for a large proportion of the oral mucosa surface area [95]. In contrast, the pilot LED device used in this study was inserted within the mouth and had a semi-circular configuration which provided a uniform light exposure in close apposition to most of the oral mucosa. This configuration compensated for the energy attenuation observed with LED sources. The values of the energy density that were utilized in our clinical pilot study and clinical trial conducted by Schikora et al., 2020 [89] were higher than the energy density range of 18–36 J/cm^2^ successfully employed in a case series study reported by Tardivo et al., 2005 [96] to treat several superficial cancers and infections utilizing MB-PDT. The PDI treatment parameters are outlined in Table 2.

For the PDI phase of treatment, Figure 2 shows the device placement below the nostrils (photo A) and inserted into the oral cavity (photo B) positioned to irradiate the entire oro-pharynx region after topical MB solution was applied to these areas. For the administration of PDI to the oro-pharynx, the teeth were held widely apart and the device was placed between the upper and lower teeth in order to minimize blockage of the light energy by the teeth and gums, thereby maximizing light exposure to the mucosal surfaces of both the mouth and throat.

**Figure 3:**
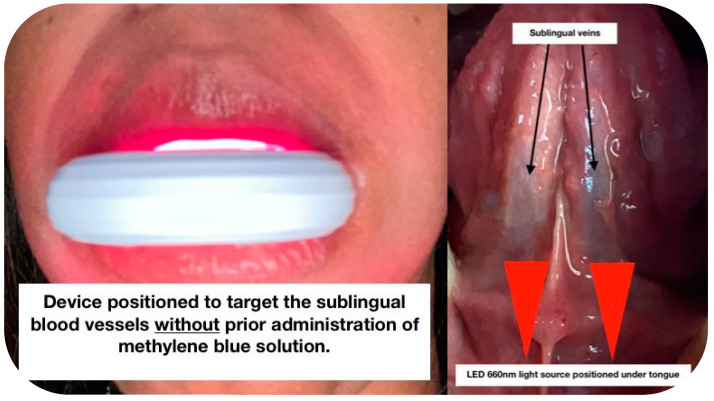
Illustrates the position of the λ 660 nm LED device targeting the sublingual vessels with PBM photonic energy.

For the administration of PBM therapy, the device was positioned to treat the ventral surface of the tongue without applying MB solution prior (Figure 3). The range of the required light energy density for PBM was much lower than the energy density required to effectively administer PDI (Table 3). In fact, higher energy density of PBM can have inhibitory effects. This is referred to as the bi-phasic dose response whereby lower doses (energy density) are more effective at stimulating and repairing tissues than higher energy density [97]. Peak PBM responses generally observed at doses of 3–6 J/cm^2^ for superficial PBM applications.

However, in administering PBM to the blood circulation, the fact that the blood components are constantly moving past the light source must be considered in calculating the energy density. Blood flow rates vary widely depending on vessel type, location, size and other influences. The sublingual veins are the major target of sublingual PBM. They are prominent veins extending dorsally from the tip to the root of tongue and drain directly into the large internal jugular vein. Based on sublingual vein blood flow rates [98], the blood components may only be stimulated for a minute or less, especially since PBM tends to increase the local blood flow rates. Therefore, the calculated value of administered energy density was approximately 4.9 J/cm^2^ based on 60 s exposure.

When administering PBM, the pilot study device was positioned to treat the undersurface of the tongue without applying MB solution prior (Figure 3).

#### 2.2.5. Patient Education

Patients were provided with a web link that provided detailed instructions on how to carry out treatments including brief training videos. This was reinforced by instructions given by phone and photographic confirmation of proper device placement sent to the treating physician.

### 2.3. Description of the Treatment Protocols

The initial goal of our study was to confirm the results of the German COVID-19 MB-PDI clinical study conducted by Schikora et al., 2020 [89] which recruited 1200 subjects. We aimed to confirm these results starting with a pilot study in our local population in the Bahamas. The plan was to assess the response of one to two ONPDI treatment sessions with additional supportive therapy in the event of late presentation as this approach appeared to be an effective treatment for COVID-19 up that point. However, to our surprise and dismay from the outset of the pilot study, we were suddenly faced with an entirely different clinical presentation. Patients were presenting with extremely high viral loads and rapidly progressive disease consistent with the Delta variant. Although we did not have access to genetic sequencing documentation, the Delta surge was also widely documented during the same period in the United States, which accounts for more than an 82% of visitors to the Bahamas [99], so it is reasonable to assume that similar variant profile would be expected in these neighboring countries.

The protocol was therefore progressively adapted and fine-tuned over the course of the eight documented exploratory cases, in order to achieve more efficient viral clearance and clinical recovery. This culminated in an establishment of our standardized three-phase Luminnova Upper Respiratory Protocol (LURP) by the end of this pilot series (Table 4). Of note, a separate case series employing LURP was subsequently documented, with even more promising results. This will be published separately.

#### 2.3.1. Phase I: Oral-MB

MB capsules were initially administered orally in a dose range of 1.5 to 2 mg/kg 12-hourly. We subsequently transitioned to 8-hourly based on observed patient responses, with a maximum dosage of 200 mg. MB capsules were withheld if there were any potential drug interactions or medical contraindications. Patients were not screened for Glucose-6-phosphate dehydrogenase (G6PD) deficiency, as studies have shown that the dosing level that is commonly used can be safely administered to both adults and children with G6PD [30,100].

#### 2.3.2. Phase II: Sublingual LED-PBM Therapy

PBM was introduced as an additional measure to address the pronounced hyper-inflammatory and hypoxic state encountered in the majority of the subjects based on their presenting symptom profiles. The advantage of this non-pharmacologic measure is that there are no medical or pharmaceutical contra-indications and it was very easy to incorporate from a logistical and safety perspective. The LED-PBM phase of therapy was achieved by inserting the LED device directly under the tongue with the ends of the device lateral to the teeth and gums. It must be emphasized that PBM treatments were administered without prior application of topical MB solution. This step was carried out just prior to the PDI phase in order to minimize the presence of MB staining in the mouth which could lead to a photodynamic rather than a photobiomodulation response. We found that, apart from the superior surface of the tongue, MB was quickly cleared from the oral cavity by saliva flow, certainly within 4 h.

#### 2.3.3. Phase III: Local Oral/Nasal PDI (ONPDI)

Local ONPDI was administered to target SARS-CoV-2 virus in the epithelial lining of the salivary glands, oral cavity, nasal and throat passages. The LED device that emits red light (λ 660 nm) was utilized to activate 1% MB solution, which was topically administered on the mucosal surfaces within the oral and nasal cavities.

Patients were instructed to gently apply the MB solution to the nasal mucosa with a cotton tipped swab and wait 10 min to allow absorption before activating the MB solution with the LED device. While waiting for absorption to occur at the nasal mucosa, the oral cavity was rinsed with 7.5 mL of MB solution for 1 min. PDI treatment was carried out immediately after rinsing with MB in order to avoid the solution being washed away or excessively diluted by saliva. The device was held in place (Figure 2) for 5 min. As the MB solution was heavily absorbed by the upper surface of the tongue, patients were strictly instructed to hold the tongue upwards as far away from the device as possible for the full duration of the oral cavity PDI treatment.

Once the oro-pharynx PDI was completed, subjects then irradiated the nasal passages for 5 min with the LED device also shown in Figure 2.

A solution was provided to rapidly remove MB staining from the lips and teeth after the treatment, but most patients did not use it until the end of their treatment course as they were in isolation, and this was not considered a high priority considering that they were significantly unwell. This option was more extensively used when the treatment had been applied as a prophylactic measure by asymptomatic close contacts of COVID-19 patients.

Of note, the local ONPDI phase III effect may also have been augmented by activation of orally administered MB capsules which could potentially achieve therapeutic concentrations suitable for a photodynamic response within the vascular network of the nasal and oral passages. This phase of treatment may have also conceivably resulted in a whole body systemic PDI component with activity against transient episodes of viraemia.

### 2.4. One-Year Follow Up

In view of the high prevalence of post-acute sequelae of SARS-CoV-2 disease (PASC) and repeat infections, subjects were followed-up after a period of one year. They were asked three questions listed below:

1. Have you had any persistent symptoms after your initial COVID-19 infection which lasted for a period 1–3 months or greater?
2. Have you had any recurrence of COVID-19 symptoms after your initial infection?
3. Have you had a positive COVID-19 test after your initial infection?

## 3. Case Description and Results

### 3.1. Subject #1

A 61-year-old Afro-Caribbean female with a history of recurrent bronchitis, BMI 31.1 and no history of previous COVID-19 vaccination, presented three days after symptom onset on 22 June 2021. She commenced the following treatments one day prior to Luminnova COVID-19 protocol: Bisolvon, Paracetamol, Ivermectin, Zithromax and Aerius.

#### 3.1.1. Results of the Initial Treatment Series

Two ONPDI treatments were administered. Her symptoms improved initially, but after two days her symptoms escalated once again and oral-MB was therefore prescribed, as a supportive measure at 1 mg/kg twice daily. We noted an improvement after each dose with flares after about 6–8 h so the dosage frequency was increased to 8-hourly.

**Figure 4:**
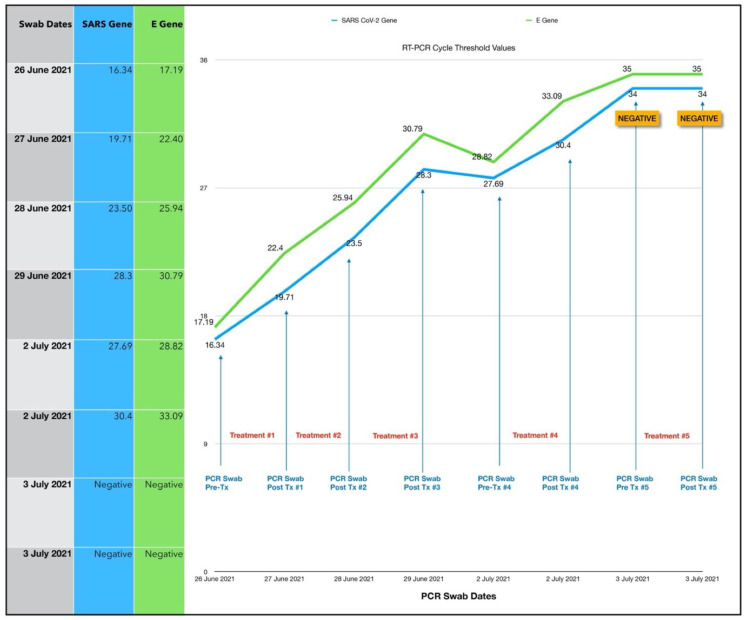
Shows subject one serial CT value.

Four days after commencing oral/nasal PDT treatments, we were able to obtain PCR CT values. To our dismay these were E gene 17.19 and SARS-CoV-2 gene (nucleocapsid/RdRp genes) 16.34. Based on this very high viral load and the fact that diarrhea was a prominent symptom, we suspected the Delta variant. At this point, we discovered that although she had been given written, pictorial and verbal instructions, she had not placed the device correctly in the mouth and therefore had not had effective photodynamic treatments. By the time we obtained CT results, she was at Day 8 of her illness. A second series of treatments were then carried out with proper device placement, which is explained in Section 2.3.3 and illustrated in Figure 4.

#### 3.1.2. Results of the Second Treatment Series

ONPDI treatments were administered daily for three consecutive days. There was a gradual rise in the CT levels consistent with a reduction in the viral load. It can be argued that the viral load could have been gradually reducing at this point even without treatment. However, when the treatments were paused for 2 days, the CT values decreased and then increased again upon resumption of the protocol, signaling that the viral load reduction resulted from the treatment. Furthermore, the progress in terms of symptoms also followed the same pattern of improvement, relapse and further improvement upon resumption of treatment.

**Figure 5:**
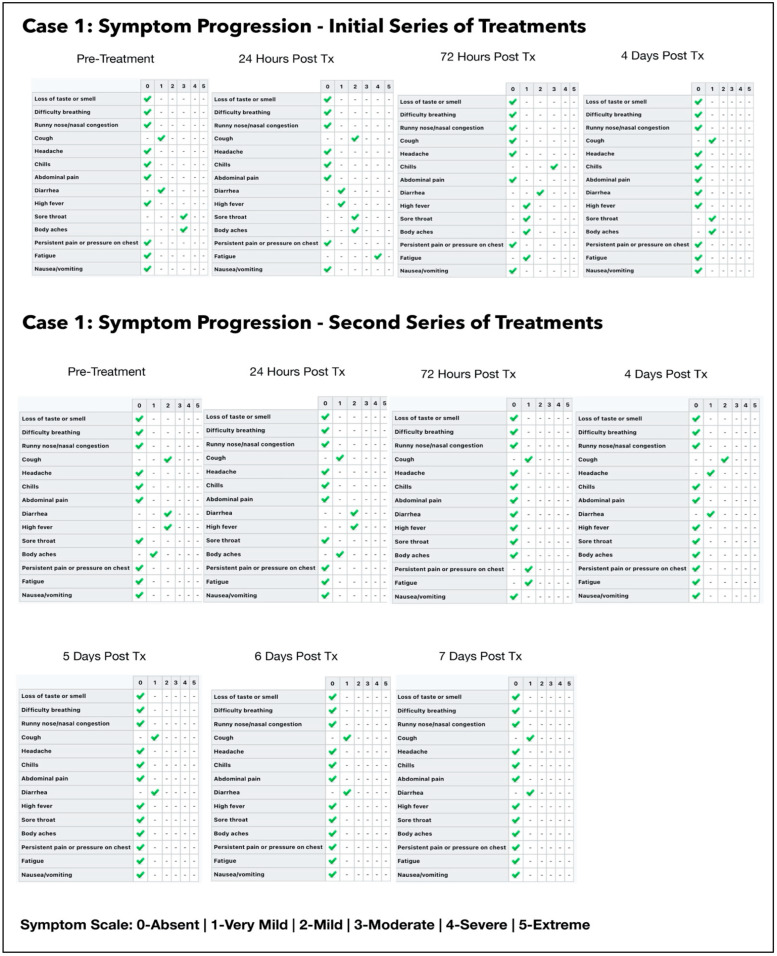
Shows the symptom progression on the initial and second series of treatments. The recorded symptoms were loss of taste and smell, difficulty in breathing, runny nose/nasal congestion, cough, headache, chills, abdominal pain, diarrhea, high fever, sore throat, body aches, persistent pain or pressure on chest, fatigue, nausea/vomiting. The symptom scale ranges from 0–5, where 0 score represents absence of symptoms and 5 score represents extreme symptoms.

In order to gain a better understanding of the treatment response and the optimal timing for the follow-up swabs, we decided to perform paired swabs immediately before and after the treatments on two consecutive days. The paired CT value results on the 2 July 2021 confirmed an increase in the CT value immediately after the treatment. Interestingly, the CT value treatment that was carried out on the next day (3 July 2021) had increased to the negative range even prior to the treatment. The difference in these readings may be related to the presence of non-viable viral RNA, which had cleared by the following morning. A summary of the subject’s symptoms progression is shown in Figure 5.

#### 3.1.3. Estimated Viral Load Reduction (EVLR)

Based on the EVLR calculation, there was a 207,000-fold reduction in viral load between Day 0 when the second treatment series was commenced and Day 7 when the negative PCR test was obtained.

#### 3.1.4. One Year Follow Up

Subject one reported that she had no post-acute sequelae of SAR-CoV-2 (“long COVID”) symptoms, recurrence of symptomatic COVID-19 re-infection or subsequent positive test for SARS-CoV-2 after recovering from the initial infection (Table 5).

### 3.2. Subject #2

A 55-year-old Afro-Caribbean male with a history of hypertension and who is a cigarette smoker, BMI 34 with no history of previous COVID-19 vaccination, presented 5 days after symptom onset on 6 July 2021. The treatments that he received prior to commencing the oral/nasal PDT were as follows: Gravol, Aerius, Levofloxacin, Aeroflux and Vitamin C. The subject had travelled to a funeral in the USA where other close relatives had tested positive for COVID-19, including his sister (Subject #1). However, he had two negative COVID-19 test results. He was receiving treatment for a bacterial pneumonia, but his condition had become progressively worse, and he was very weak. He stated that the symptoms were “*the worst he had ever experienced in his entire life*”.

**Figure 6:**
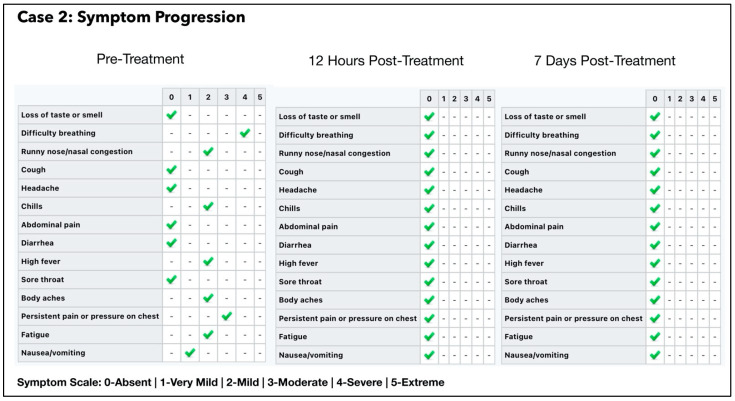
Shows symptom progression of subject two.

A repeat RT-PCR test was carried out which was positive for COVID-19 with a CT value of 22.12 E gene/20.17 SARS gene. Intravenous fluid was administered at the subject’s home along with a single ONPDI treatment late at night on 6 July. The patient made a remarkable recovery by the next morning and refused additional PCR tests, as he declared that he had fully recovered (Figure 6). At the Day 7 follow up call the patient later reported that he had experienced slight fatigue for a few days after treatment, but felt completely well at Day 7. A summary of symptom progression initially and after treatment is shown in Figure 6.

#### One-Year Follow Up

Subject two reported that he had no post-acute sequelae of SAR-CoV-2 (“long COVID”) symptoms, recurrence of symptomatic COVID-19 re-infection or subsequent positive test for SARS-CoV-2 after recovering from the initial infection (Table 5).

### 3.3. Subject #3

A 40-year-old Afro-Caribbean male who had no significant past medical history with BMI of 31.7 and no history of previous COVID-19 vaccination, presented 5 days after symptom onset on 6 July 2021. The treatments prior to commencing the oral/nasal PDI included Hydroxychloroquine, Theraflu, zinc and vitamin D3.

The treatment regimen was as follows:

1. One session per day of ONPDI: on Days 0 to 2.
2. Three sessions of ONPDI on Day 3.
3. Oral-MB was administered from 6 July to 10 July 2021 and PBM therapy was administered from 7 July to 10 July 2021.

The subject did not initially admit that he had been taking hydroxychloroquine. A relative had been prescribed this the year before with a great clinical benefit. Hence, he had self-treated, but the symptoms were significantly worsening, so he sought assistance with the Luminnova COVID-19 protocol.

**Figure 7:**
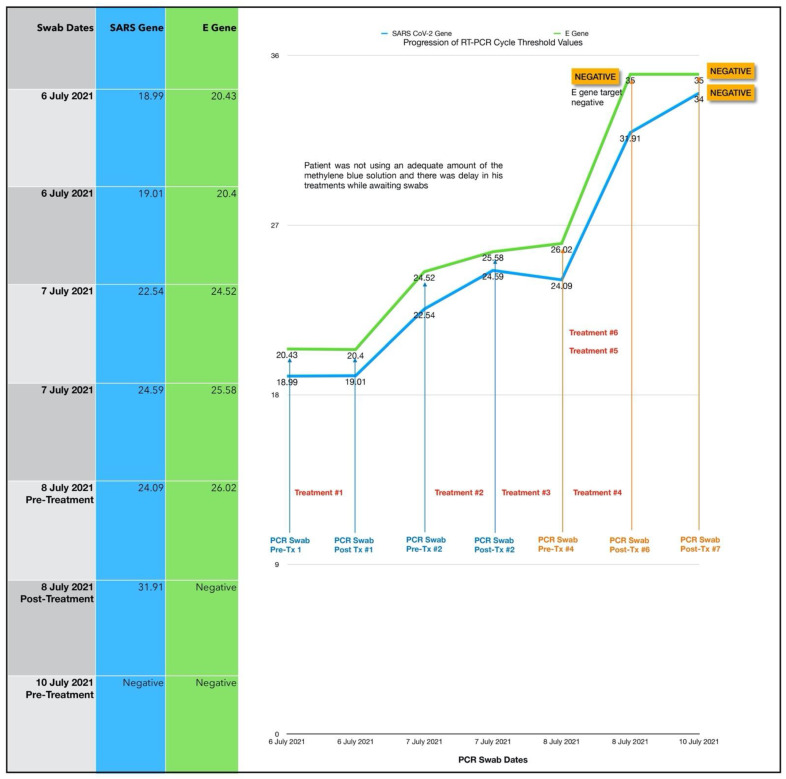
Shows the serial CT values of subject three.

In response to complaints about discomfort from nasal swabbing the technique of obtaining samples was adjusted after the initial four paired samples (Figure 7-blue arrows). He was asked to blow his nose prior to taking the sample which was taken more anteriorly in the nasal passages (Figure 7-red arrows). It appears that was actually more sensitive because this test result indicated a reduction in the CT. value (Pre-Treatment #4 swab) despite significant improvement symptoms at that point. We used this method on all subsequent pilot study swabs as shown in Figure 7.

**Figure 8:**
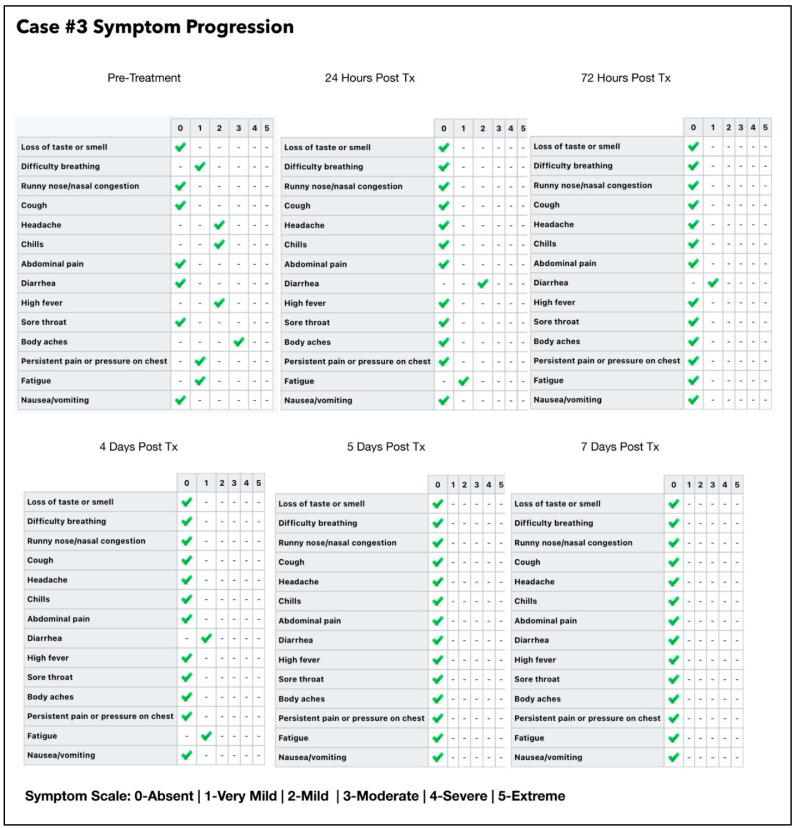
Shows the symptoms progression of subject three.

The response in CT values was initially slower than case #1 which may have resulted from a delay in the treatments while waiting for his PCR results and an incorrect treatment technique (inadequate amount of MB solution used) in this case. More importantly, based on the response observed after each treatment session, it was decided to administer three-treatment sessions in 1 day in order to achieve a more significant reduction in the viral load. PBM therapy was also added to the regimen in an attempt to bring about a greater symptom response in terms of ongoing diarrhea and chills which had persisted 24 h after commencing our treatment protocol (Figure 8).

Once the accelerated treatment schedule and other changes were introduced, there was excellent clinical progress. There was a steep increase in the CT values (indicating a marked reduction in the viral load) and a marked reduction in symptoms. In fact, the E gene target was negative immediately after the third treatment session carried out on Day 3 and both gene targets were negative by Day 4 with no additional ONPDI treatments. By Day 7, he resumed heavy manual tasks with no difficulties.

#### 3.3.1. Estimated Viral Load Reduction (EVLR)

Based on the EVLR calculation, there was a 32,995-fold reduction in viral load between Day 0 when treatment was commenced and Day 4 when the negative PCR test was obtained.

#### 3.3.2. One Year Follow-Up

Subject three reported that he had no post-acute sequelae of SAR-CoV-2 (long COVID) symptoms, recurrence of symptomatic COVID-19 re-infection or subsequent positive test for SARS-CoV-2 after recovering from the initial infection (Table 5).

### 3.4. Subject #4

A 38-year-old Afro-Caribbean female who had no significant past medical history, BMI 31.3 and no history of previous COVID-19 vaccination, presented 3 days after symptom onset on 11 July 2021. The treatments prior to the Luminnova COVID-19 protocol were as follows: hydroxychloroquine, Cetamol Cold and Flu, zinc and vitamin D3.

The treatment regimen was as follows:

1. Three sessions of topical oral/nasal PDT on Days 0 and 1.
2. Two sessions of topical oral/nasal PDT on Day 3.
3. Oral MB and sublingual PBM 8-hourly Day 0 to Day 2.

**Figure 9:**
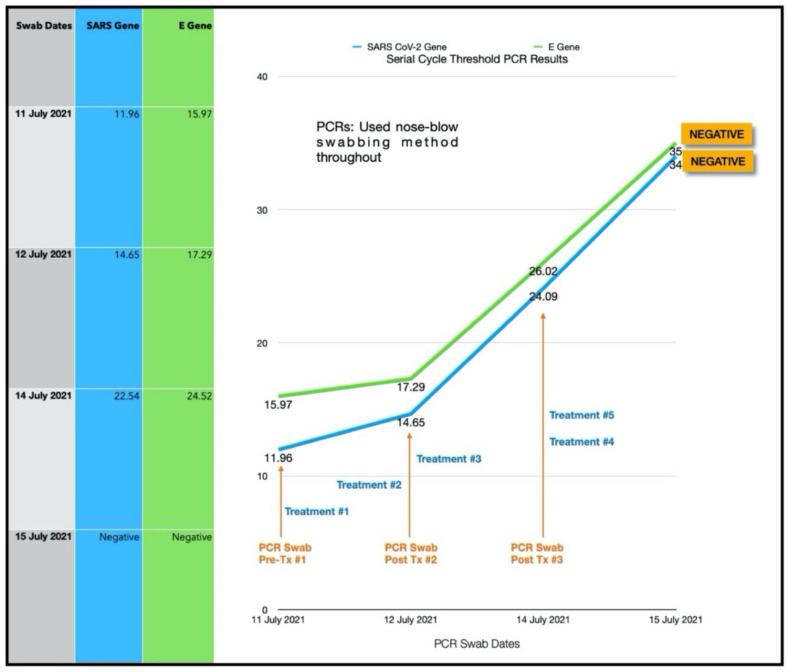
Shows the serial CT values of subject #4.

**Figure 10:**
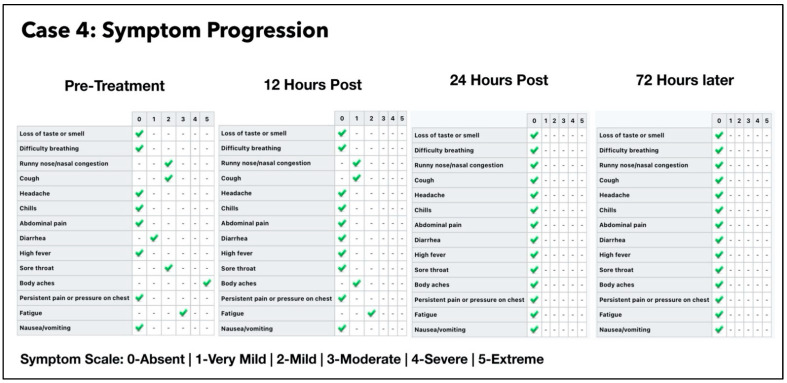
Shows the symptom progression of subject four pre-treatment, 12 h, 24 h and 72 h post-treatment based on symptom scale ranging from 0–5.

The subject was in the same household as Subject three. Of note, she had taken hydroxychloroquine prior to starting the protocol and her treatment was commenced at an earlier stage of the infection than Subject three (3 days after symptom onset). She nevertheless had very low CT values and was very unwell just prior to the treatment, as shown in Figure 9 and Figure 10. Based on the experience that we had gained from the earlier cases, the treatment schedule was accelerated. Three treatment sessions were administered in the first 24 h. The improvement in clinical response and the viral load reduction were rapid and RT-PCR was confirmed negative by Day 4.

#### 3.4.1. Estimated Viral Load Reduction (EVLR)

Based on the EVLR calculation, there was a 4,312,220-fold reduction in viral load between Day 0 when the treatment series was commenced and Day 4 when the negative PCR test was obtained.

#### 3.4.2. One Year Follow-Up

Subject four reported that she had no post-acute sequelae of SAR-CoV-2 (“long COVID”) symptoms, recurrence of symptomatic COVID-19 re-infection or subsequent positive test for SARS-CoV-2 after recovering from the initial infection (Table 5).

### 3.5. Subject #5

A 42-year-old Afro-Caribbean male with no significant past medical history, BMI of 32.5 and no history of previous COVID-19 vaccination, presented 5 days after symptom onset on 16 July 2021. The subject had not received any treatment prior to commencing the Luminnova COVID-19 protocol.

The Treatment Regimen was as follows:

1. Number of ONPDI treatments prescribed 8-hourly.
2. Oral MB-prescribed 8-hourly, but this treatment schedule was not maintained.

**Figure 11:**
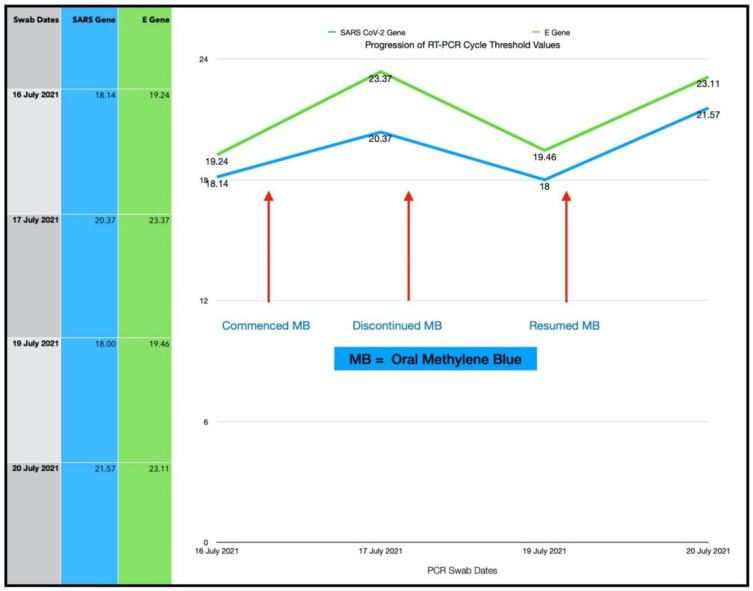
Shows the serial CT progression of subject five.

**Figure 12:**
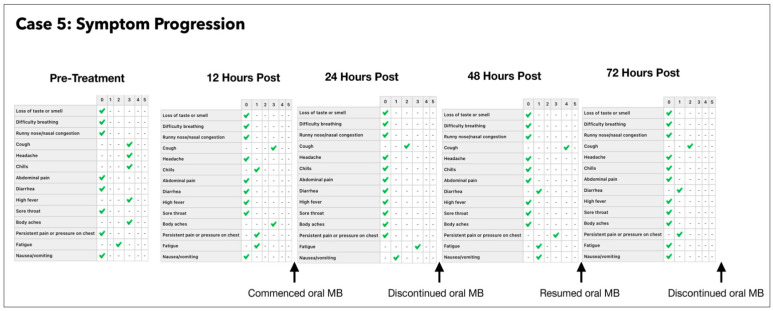
Shows the symptom progression of subject five pre-treatment, 12 h, 24 h and 72 h post-treatment based on symptom scale ranging from 0–5.

The subject was initially commenced with only ONPDI treatments, but oral-MB was also advised once the high viral load was confirmed. He was poorly compliant due to nausea. The authors believe that nausea was most likely secondary to the infection and the oral-MB capsules. Reduction in both viral load and symptoms were noted 24 h after commencing the treatment regimen including oral MB. At approximately 36 h, MB was temporarily discontinued. The third PCR at 72 h revealed that the viral load had increased once again and his symptoms had worsened. Upon re-commencing MB once again, there was a reduction in the viral load by Day 4 (Figure 11) and gain in the clinical improvement (Figure 12). However, he attributed this improvement to new supplements which he had commenced on Day 4 and opted to discontinue the Luminnova protocol. Unfortunately, despite continuing the new supplements his condition worsened after discontinuing the Luminnova protocol and he was admitted to hospital a few days later. He received intravenous fluids and supplemental oxygen in hospital, but no mechanical ventilation was required.

#### One Year Follow-Up

Subject five reported marked PASC or “long COVID” symptoms for 2–3 months after his acute infection including weakness, difficulty breathing and postural dizziness (Table 5) He did have a repeat infection with a positive SARS-CoV-2 test 5 months after his initial infection but experienced mild symptoms only at that point.

### 3.6. Subject #6

A 31-year-old Caucasian female who had no significant past medical history was referred by a colleague for treatment with the Luminnova protocol, as she was experiencing progressively worsening symptoms, due to COVID-19 infection. She was pregnant at 8 months gestation at the time of presentation with BMI of 27.6 (pregnant). She had no history of previous COVID-19 vaccination and presented five days after symptom onset on 18 July 2021. The treatments prior to the Luminnova COVID-19 protocol were Acetaminophen and antenatal supplements. The subject’s obstetrician was consulted, and approval was given to proceed with ONPDI. Despite orally ingested MB having been safely used in rare instances in late pregnancy [101], this was strictly avoided in her case.

The Treatment Regimen was as follows:

1. ONPDI:
  - First course of eight treatments; Day 0 to Day 2.
  - Second course of eight treatments; Day 4 to Day 6.
2. Sublingual PBM; Day 4 to Day 7.
3. No oral-MB was administered as the patient was pregnant.

**Figure 13:**
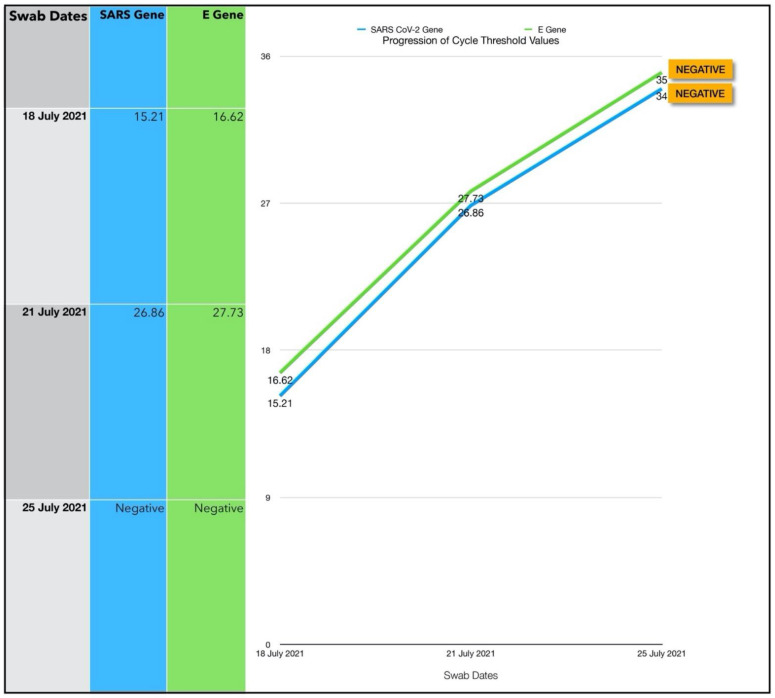
Shows the serial CT values of subject six.

Improvement in her symptoms was observed on commencing ONPDI, although at a much slower rate than previous cases (Figure 13). The challenge as with many other COVID-19 cases during this period, was that she was at home alone and became progressively more tired, dehydrated and was not eating adequately. It was therefore decided that she should be assessed and supported in a hospital setting, as a precaution. She was referred to a local emergency room with labored breathing, coughing ++ with secondary chest pain, weakness and insufficient hydration.

The subject was assessed at the Emergency Room (ER) of her local hospital on Day 3 of the Luminnova protocol and chest imaging was carried out. The ER team concluded that her condition was stable and did not warrant admission. Therefore, she was discharged the following day. Repeat CT values from a PCR swab were obtained on Day 3, but were not available on Day 4; however, they indicated that her viral load had reduced significantly even before attending the ER.

**Figure 14:**
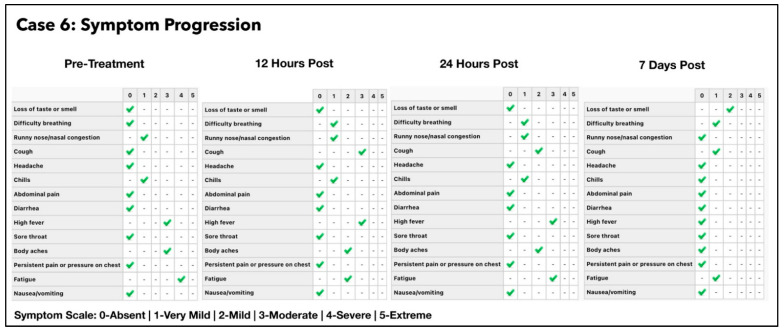
Shows symptom progression of subject six pre-treatment, 12 h, 24 h and 72 h post-treatment based on symptom scale ranging from 0–5.

As the option of hospital support was unavailable, we continued ONPDI and added sublingual PBM therapy along with nutritional supplements at home on Day 4. After the brief period of rest and re-hydration in the ER, there was much better response to the treatments administered as shown in the symptom progression chart of Figure 14. She tested negative on RT-PCR by Day 7 and made a good recovery. She returned to work 10 days later with a full workload in a demanding health care setting and later delivered a healthy baby.

#### 3.6.1. Estimated Viral Load Reduction (EVLR)

Based on the EVLR calculation, there was a 450,000-fold reduction in viral load between Day 0 when the second treatment series was commenced and Day 7 when the negative PCR test was obtained.

#### 3.6.2. One Year Follow-Up

Subject six reported that she had no post-acute sequelae of SAR-CoV-2 (“long COVID”) symptoms, recurrence of symptomatic COVID-19 re-infection or subsequent positive test for SARS-CoV-2 after recovering from the initial infection (Table 5).

### 3.7. Subject #7

A 47-year-old Afro-Caribbean female with no significant past medical history, BMI 29.2 and no history of previous COVID-19 vaccination, presented 2 days after symptom onset on 21 July 2021. The subject has not received any treatment prior to the Luminnova COVID-19 protocol.

The treatment regimen was as follows:

1. Topical ONPDI-course of eight treatments, Day 0 to Day 2
2. No oral-MB administered as patient presented early and had mild symptoms

**Figure 15:**
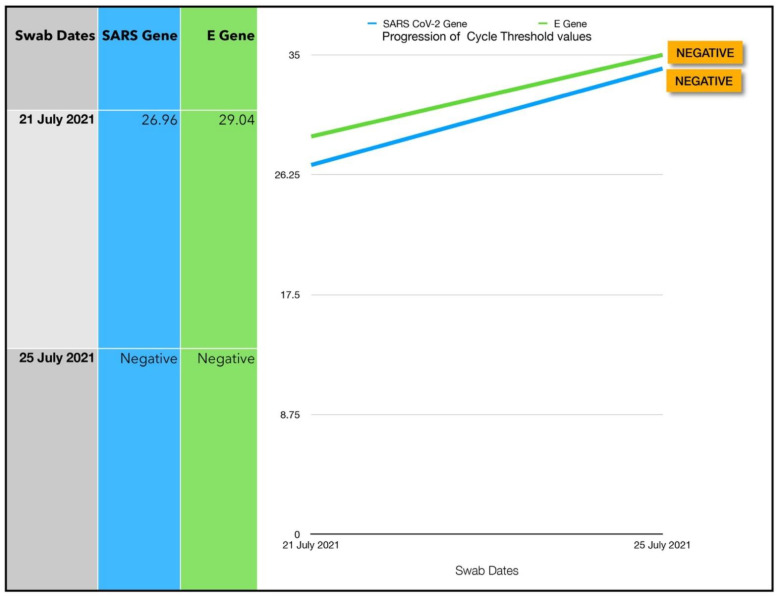
Shows the serial CT of subject seven.

The patient was referred by a close contact who had been treated with this protocol. As she presented early and only had a moderately high viral load, only ONPDI was administered. She experienced mild symptoms only and made a rapid recovery. RT-PCR was repeated on Day 4 and confirmed negative (Figure 15).

#### 3.7.1. Estimated Viral Load Reduction (EVLR)

Based on the EVLR calculation, there was a 128-fold reduction in viral load between Day 0 when the treatment was commenced and Day 4 when the negative PCR test was obtained.

#### 3.7.2. One Year Follow-Up

Subject seven reported that she had no post-acute sequelae of SAR-CoV-2 (“long COVID”) symptoms, recurrence of symptomatic COVID-19 re-infection or subsequent positive test for SARS-CoV-2 after recovering from the initial infection (Table 5).

### 3.8. Subject #8

A thirty-eight-year-old Afro-Caribbean male with no significant past medical history. His body mass index (BMI) was 32.5 and no history of previous COVID-19 vaccination, presented 3 days after symptom onset on 21 July 2021. There was no treatment prior to commencing the Luminnova COVID-19 protocol. The treatment regimen was based on a three-phase protocol as follows:

1. Oral-MB capsules.
2. Sublingual PBM.
3. ONPDI.

The initial treatment regimen was a total of eight treatments given every 8 h, Day 0 to Day 2, whereas the repeat treatment regimen was a total of eight treatments given every 8 h, Day 4 to Day 6.

**Figure 16:**
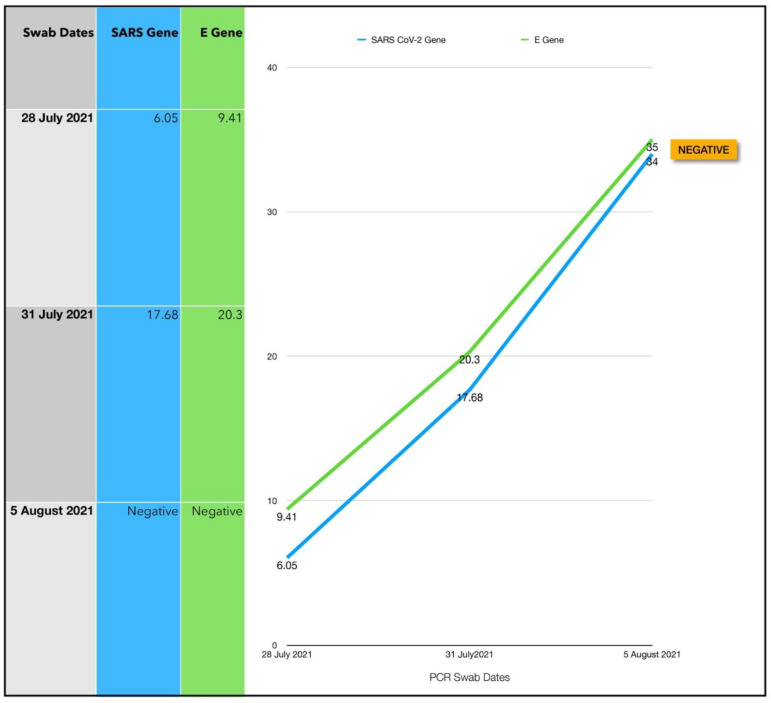
Shows the serial CT values of subject eight.

Subject eight presented with prominent symptoms and an extremely high viral load. The initial CT value was 6.05/9.41 (Figure 16). These results were confirmed by the lab with a repeat assay. It was clear that an aggressive approach would be required to reduce the viral load, provide effective supportive therapy and to mitigate his risk. PBM was therefore added to the treatment regimen from the outset along with ONPDI and oral-MB. All three phases of the treatment protocol (oral-MB, PBM and PDI) were administered 8-hourly from the outset.

**Figure 17:**
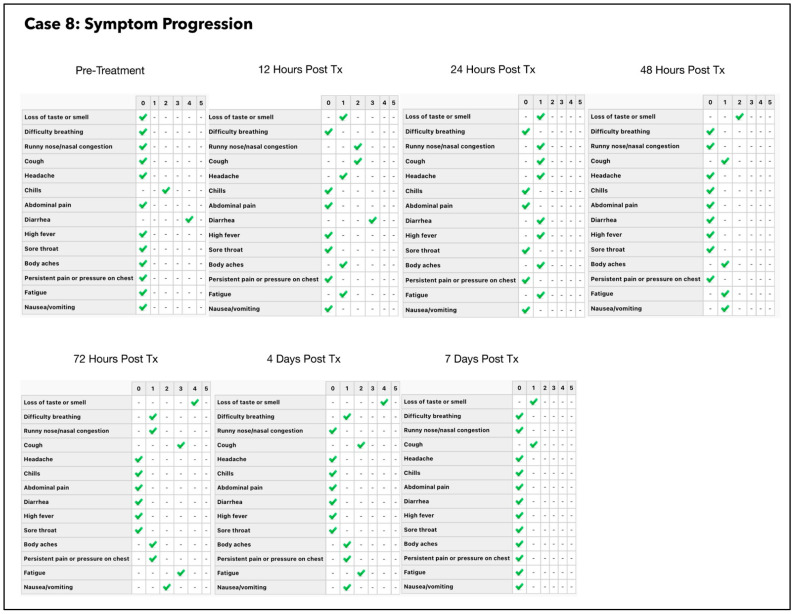
Shows the symptom progression of subject eight pre-treatment, 12 h, 24 h and 72 h post-treatment based on symptom scale. ranging from 0–5.

This treatment protocol regimen proved to be very successful. Repeat PCR at Day 3 confirmed an 11-point increase in the CT values, indicating a 2000-fold decrease in the viral load within 72 (Figure 15). This was significant as the treatment was commenced at the rapid replication phase of the infection. Treatment was paused for 2 days between Day 3 and Day 4 while awaiting the PCR result. This coincided with an exacerbation in symptoms. After a close COVID-19 contact tragically died, he complained of feeling very weak and faint and attended a local ER, where he received intravenous (IV) fluids. He was discharged a few hours later as there were no significant clinical findings apart from dehydration. In retrospect, treatments should have been continued considering the massive initial viral load present. Once treatment resumed, the symptoms started to improve once again and by day 7, the symptoms were minimal (Figure 17). According to the patient “besides my loss of taste I have completely turned around and I feel great. Almost back to normal.” PCR testing was delayed until day 8 for logistical reasons. At this point, the PCR was confirmed negative.

#### 3.8.1. Estimated Viral Load Reduction (EVLR)

Based on the EVLR calculation, there was a 259-million-fold reduction in viral load between Day 0 when the treatment was commenced and Day 8 when the negative PCR test was obtained.

#### 3.8.2. One Year Follow-Up

Subject eight complained of mild persistent symptoms which he described as “mucous on his chest”, but no additional symptoms such as fatigue, cardiac symptoms, brain fog or other neurological symptoms. There was no recurrence of symptomatic COVID-19 infection or subsequent positive test for SARS-CoV-2 (Table 5).

## 4. Discussion

Our pilot study of eight cases (eight subjects) was supported by objective data (serial PCR CT measurements) and self-reported outcome questionnaires completed by each subject. This study provides an early indication that the combination of local ONPDI, sublingual PBM and orally administered MB may be an effective means of managing even challenging COVID-19 disease in the home environment. Seven subjects were of Afro-Caribbean ethnicity and one subject was Caucasian. To the best of the authors’ knowledge, this is the first COVID-19 clinical case series published in an Afro-Caribbean population. Subject ages were between 31–61 years. Most subjects were at a higher risk for poor COVID-19 outcomes. One subject was pregnant (8 months gestation) and the majority had BMI of 31 or greater. Apart from one subject who had moderately high viral load, viral loads at presentation ranged from very high to extreme in one subject. Despite these risk factors, expedited clinical recovery was observed in parallel with very rapid of SARS-CoV-2 viral clearance. The median negative PCR test and clinical recovery time of the seven subjects who completed the treatment was 4 days. This underlines the significance and the effectiveness of our treatment protocols even in the context of high BMI, very high viral loads and suspected Delta variant COVID-19 disease. We have outlined the key points of our results and their significance below.

### 4.1. Assessment of Treatment Response and Outcomes

Our pilot study (Figure 4, Figure 5, Figure 6, Figure 7, Figure 8, Figure 9, Figure 10, Figure 11, Figure 12, Figure 13, Figure 14, Figure 15, Figure 16 and Figure 17) offered useful insights into the degree to which each treatment session can reduce the viral load. A summary of the results is provided in Table 5.

There were progressive increases in CT values and a parallel reduction in symptoms with successive treatment sessions. Moreover, a slowing or reversal in the trend of these improvements was also observed when the treatments were paused temporarily, and continued improvement was then noted upon resumption of the protocol (e.g., subjects: one, three and five). We therefore concluded that responses observed were likely due to the treatments administered.

Large reductions in viral loads were documented over very short periods (Table 5). This was observed in all subjects apart from subject five who was not able to fully comply with the treatment protocol due to nausea. Nevertheless, the observed changes in Subject five provided greater insight into the contribution of orally ingested MB in reducing both the viral load and symptoms. We noted a correlation between the use of MB and progression of the viral load along with symptom severity in this subject (Figure 11 and Figure 12).

We were unable to document serial CT changes for Subject two as he refused repeat PCR and insisted that he had fully recovered 24 h after a single ONPDI session. Notwithstanding the lack of laboratory confirmation, there was an excellent clinical recovery and good long-term outcomes as indicated by his questionnaire responses (Figure 6) and 1 year follow up (Table 6).

The CT values observed in the other six subjects suggests that these interventions may have great potential in high viral load COVID-19 disease. The most striking response was noted for subject eight who presented with extremely low CT values (6.05/9.41). A more aggressive treatment approach was taken with the aim of expediting clearance of the SARS-CoV-2 virus and protecting the lungs and other organs. The treatment schedule was therefore accelerated. The dramatic reductions in viral load (259-million-fold) and rapid recovery in this subject with a high BMI and suspected Delta variant COVID-19 was highly encouraging. We, therefore, adopted the protocol administered to this subject as a standardized approach going forward.

Notable reduction in symptoms were observed within 12–24 h of commencing treatment in all the subjects apart from subjects; one and six. The slow response observed initially for Subject 1 was due to incorrect device placement. Once this issue was addressed there was good clinical response. Subject six was not administered oral MB as she was pregnant (8 months gestation). Although there was a marked reduction in viral load, symptomatic response was significantly slower in this subject in comparison to the other subjects. This may have been at least partially due to pregnancy. However, a rapid clinical response to MB in other subjects along with the regression in symptoms on discontinuing MB in Subject five, suggests that withholding MB may also have been a factor in Subject six. Despite the fact that MB had to be avoided, significant symptom reduction, including reduced cough and weakness were noted for Subject six following the introduction of sublingual PBM on Day 4. Overall, her response was encouraging considering that systemic pharmacological agents were avoided due to pregnancy. Summary of the results illustrated in Table 6.

We also observed that there was a correlation between CT levels and the severity of symptoms. Contrary to prevailing opinion, the CT values appeared to be a highly predictive tool in assessing clinical prognosis and progress in the pilot cases during the Delta phase of the pandemic. This was likely aided by the use of a single laboratory for PCR results. However, the cost and 12–24 h delays in getting PCR results at times presented a challenge in utilizing these tests to support clinical decision making.

### 4.2. Adverse Effects

Adverse effect data were elicited via daily online follow up questionnaires and telephone conversations. Subjects were specifically asked to describe in as much detail as possible if they experienced any difficulties or adverse effects from the treatment with each follow up questionnaire completed. The responses obtained indicated that the treatment protocol was well tolerated. There were a few reports of minor issues, but no serious adverse events were reported.

Subject five was unable to consistently comply with and did not complete the recommended protocol. He had complained of nausea on taking oral MB. This improved somewhat once his dosing schedule was adjusted to space the oral MB dosing without reducing the total daily dosage. However, this was still a challenge and subsequently led him to abandon the treatment protocol.

Three subjects (one, six and eight) had to attend the ER due to dehydration. In these three cases, in-patient hospital admission was not considered necessary as no significant lung or other organ impairment was detected. Dehydration was at least partially due to diarrhea, nausea and vomiting which were commonly associated with the Delta variant. However, oral MB was also considered a potential factor as it is almost exclusively excreted via the kidneys. Once a more concerted effort at oral hydration was emphasized, this problem was largely avoided, and oral MB was generally well tolerated.

Subject one complained of mild stinging of the throat after the first ONPDI treatment, but this had resolved in less than 30 min. This appeared to be related to gargling with the MB solution. Hence, subject one and subsequent subjects were advised to actively rinse the oral cavity, but to minimize gargling. Subjects were also instructed to hold the tongue upwards and back in order to avoid burning the upper surface of the tongue which heavily absorbed MB solution. No other study subjects complained of burning of the throat, tongue or other sites.

When employing a light-based therapy, it is important to consider the skin type of subjects treated as melanin is an active chromophore of specific wavelengths. Potential problems resulting from treatment of melanin rich sites include reduced absorption of the light energy and/or excessive heating of the target tissues. Fortunately, the mucosal surfaces of the nose and mouth do not contain melanin and therefore we did not anticipate that these issues would arise. Seven of the eight subjects were skin types VI and one subject was skin type II. The treatment protocols were demonstrably effective in all subjects who completed the protocol, indicating adequate light penetration. Secondly, the ONPDI and PBM were well tolerated overall, with no perception of heat at the treatment sites.

### 4.3. Long-Term Follow-Up

Post-acute sequelae of SARS-CoV-2 (“long COVID”) and recurrent COVID-19 infection have proven to be frequent and highly problematic issues associated with SARS-CoV-2 viral infections. Six of the seven subjects who completed the protocols reported no long COVID symptoms, recurrence of symptomatic COVID-19 infection or subsequent positive SARS-CoV-2 test results (Table 6).

Subject eight reported mild persistent chest congestion up to one year post treatment. This was perhaps not surprising as his initial viral load was the highest documented of all the patients treated with the Luminnova protocols over the course of two years. There was also a pause in his treatment over two days due to attendance at a local hospital Emergency Room due to dehydration and discharged after a few hours. Extremely high viral load and the pause in treatment may have led to a higher risk of lung injury, albeit mild. He reported no recurrence of symptomatic COVID-19 infection or subsequent positive SARS-CoV-2 test results (Table 6).

Subject five was unable to complete the protocol and was the only subject that had marked and prolonged symptoms after recovering from acute SARS-CoV-2 infection. He was also the only subject that reported recurrence of symptomatic COVID-19 infection with positive SARS-CoV-2 test results (Table 6).

These findings may indicate the need to clear SARS-CoV-2 as quickly and as thoroughly as possible, in order to avoid long-COVID symptoms. Subject 5 was also the only subject to report repeat symptomatic infection with a positive SARS-CoV-2 test. The finding that all subjects who completed the protocol had no recurrence of SARS-CoV-2 symptoms or positive tests, may also point to a durable immunity conferred in the context of these subjects. However, further studies in a larger population would be required to confirm this.

### 4.4. Logistical Considerations

In late June 2021, there was a deluge of significantly ill-COVID-19 patients in the Bahamas and elsewhere. Hospitals and acute care clinics were overwhelmed, and cross-infection was a significant concern. As a result, COVID-19 patients were advised to quarantine at home and only seek hospital intervention, if they developed breathing difficulties. Naturally this led to significant anxiety, as many patients were very unwell and were aware of a number of reports of deaths in the community. There was a large unmet need for effective early treatment and home support for these patients.

Our treatment protocol was designed to facilitate minimal contact between patients and the healthcare team and allow patients a viable option of continuing self-care at home, thereby reducing the burden on healthcare facilities. A single-person LED treatment device was customized for this purpose. This approach avoided the risk of laser eye injury and the need for on-site medical supervision. However, a significant amount of time was required to provide remote support and advice and there were communication challenges, which had to be addressed.

Subject one underlined the challenge of ensuring that subjects understood precisely how to place the device for effective treatments. In this case the device had been placed incorrectly initially resulting in inadequate clinical progress. As a result, more reliable ways of communicating this information were developed and Subject one and subsequent subjects were able to reliably carry out the treatments independently in the home setting with minimal on-site medical intervention. We therefore concluded that the treatment protocol administered was feasible provided adequate patient education was provided.

### 4.5. Evaluation of Related-Published Clinical Studies

To our knowledge, this is the fourth publication to document a clinical response to ONPDI for COVID-19 infection. The first study was a controlled clinical study of 1200 subjects carried out in Germany in early 2020, employing MB-ONPDI [89]. This study demonstrated a high degree of efficacy in addressing acute COVID-19 disease during the initial stages of the pandemic with an 83% reduction in hospital admissions and a 90% reduction in ICU admissions and death. Qualitative PCR data confirmed 100% conversion from positive PCR test for SARS-CoV-2 to a negative test after a single 30-min treatment session in the active treatment cohort.

Interestingly, we had also noted a rapid response to one to two ONPDI treatment sessions during the early phase of the pandemic when the original Wuhan strain was still dominant in the Bahamas. Treatment was administered using the same LED device used in this pilot study. We had noted that patients remained well after treatment without any recurrence of symptoms, providing that the entire household was treated. However, as noted earlier, an expanded and accelerated protocol was needed to manage the most aggressive COVID-19 disease encountered during the Delta wave (Table 4).

A smaller controlled clinical study by Weber et al., 2020 [94] employed riboflavin ONPDI for COVID-19 [94]. They similarly documented serial changes in CT values as a surrogate measurement of viral loads. It is not possible to make precise comparisons of our study results because the Weber study did not document the initial CT values and a different PCR assay was used. However, based on the serial follow up CT measurements provided; it is clear that the subjects who were treated in the latter part of 2020 had far lower viral loads than our pilot subjects who were treated in late June/July 2021 during the Delta surge in the Bahamas. Despite very high viral loads at presentation, clearance rates in our cohort appeared to be much faster than observed in the Weber study. In Weber et al., 2020 [94] study, the majority of the subjects with high viral loads (CT value < 24) had not cleared by Day 7. In contrast, all of our pilot subjects who completed the protocols cleared within 4–8 days (median 4 days) despite initial CT values mostly in the low to mid-teens and as low as six. We attribute this expedited recovery to the adaptation of the protocol with an accelerated treatment schedule and addition of orally ingested MB and PBM therapy. Furthermore, methylene blue is activated by a more deeply penetrating wavelength (λ 660 nm) than riboflavin (λ 375/λ 447 nm) [94], and thus provides deeper tissue action with potentially greater efficacy.

Another group who demonstrated that SARS-CoV-2 can be reduced to undetectable levels in the nose utilizing an established MB-PDI platform for pre-surgical infection prophylaxis [102]. However, their protocol did not target the oral cavity which is a significant reservoir for SARS-CoV-2 replication [91].

The benefits of oral-MB and PBM for COVID-19 disease have also been previously reported. Randomized controlled trials Phase I, II and III conducted by Alamdari et al., 2020, 2021 and 2021, respectively [103,104,105], have demonstrated the benefit of systemically administered reduced-MB along with vitamin C and *N*-acetyl cysteine in severely ill COVID-19 subjects in ICU. They documented impressive responses with statistically significant improvements in laboratory results (methemoglobin, CRP, LDH) and clinical status (rapid reversal of hypoxia and recovery in severely ill COVID-19 subjects) [100,101,102].

Sigman et al., 2020 [106] described notable improvements in clinical status with a series of PBM treatments using a scanning treatment device in a single case study. Oxygen saturation was increased, pneumonia severity index was markedly improved and there was a substantial decrease in C reactive protein (between 15.1–1.23 over 4 days).

Williams et al., 2022 successfully employed transdermal PBM in a non-randomized 50-person case study [107]. They confirmed a resolution in symptoms in approximately 80% of cases within 4 days and 100% within 3 weeks. Oxygen saturation was also improved in all subjects, a few considerably. These results underline the potential of PBM to make a meaningful contribution in reducing hyper-inflammation, improving oxygenation and promoting organ recovery in COVID-19 disease.

Our pilot study employed sublingual PBM as opposed to transdermal PBM due to the absence of melanin and the proximity of the target cells in the blood to the light source, a much lower fluence is needed at this site. The use of a single-person LED device also facilitated independent treatment in the home-setting with much lower potential for cross-infection between the medical team and patients. Further studies assessing the magnitude of response to sublingual and/or intra-nasal PBM used independently of other treatment interventions for COVID-19 are warranted.

### 4.6. Study Limitations

A significant limitation of our pilot study was the inclusion of only a small number of subjects in which a single, standardized protocol was not used. Nevertheless, despite these important limitations, the magnitude of viral load reduction and rapidity of the clinical response in a population who were at risk was indeed promising. This provides an early indication that these protocols may be a viable means of addressing COVID-19 disease in the community.

Inflammatory markers, basic metabolic and hematologic assessments and chest scans were not documented. Additional understanding of the immunological response and its impact on the organ systems could be obtained by including these investigations before and after use of our protocols.

As MB has independent anti-viral actions, a control arm including application of the solution without photodynamic activation would be helpful to clarify whether the progressive changes in viral load were due at least in part to the solution alone. The controlled study by Schikora et al., 2020 [89] provided clarity on this question. Negative PCR results were documented after a single treatment session accompanied by highly successful clinical outcomes in the active MB-PDI arm. In contrast, the placebo subjects had MB solution applied to the nasal and oral mucosa without photodynamic activation. In all these control subjects, a repeat PCR was still positive and they had markedly inferior clinical outcomes compared with the active group.

Although the use of three separate treatment interventions (MB, PBM and ONPDI) achieved great clinical response in our pilot subjects, a further limitation we noted is it did not provide us with a clear understanding on how much each intervention contributed to these positive outcomes. Hence, gaining a greater understanding of each intervention effect would be very beneficial in further protocol adjustments, but was not considered ethical in the context of a high-risk disease where the overwhelming priority was to expedite recovery.

Lastly, statistical analysis was not feasible in view of the evolving protocols utilized. However, based on the findings of our pilot study, a second case series using a standardized protocol was subsequently documented also during the Delta phase and will be published separately. A similar protocol is now being successfully utilized to target a series of Omicron sub-variants. The durability of this response underlines the value of PDI, MB and PBM to target COVID-19 in a non-variant specific manner.

### 4.7. Future Potential

Due to the broad spectrum of action against multiple microbial species, coupled with lack of documented evidence of microbial resistance over decades of use, PDI has the potential to target current and emerging COVID-19 variants. This is a critically important advantage of this treatment approach. Moreover, there is potential to address many other pathogens contracted via the nasal and oral mucosa, including influenza, the common cold, respiratory syncytial virus, adenovirus infections and possibly pox viruses, using these combined protocols [108,109,110].

## 5. Conclusions

The combination of oral-MB, PBM therapy and local ONPDI provided a comprehensive and effective treatment approach to at risk, high viral load associated COVID-19 disease in the pilot cases documented. Firstly, we demonstrated rapid clearance of the SARS-CoV-2 virus as evidenced by CT data. Secondly, the immune modulating effects of oral-MB and PBM therapy are both well recognized and their inclusion in our protocol appears to have offered critical benefits in managing COVID-19 with expedited recovery of the lungs and other affected organs. This approach may potentially benefit other severe infectious diseases, where morbidity and death result not only from the direct consequences of infection but where the magnitude and type of host response may be even more problematic.

The deployment of a home-based protocol offered multiple benefits both for patients and the healthcare team, including reduced contact between patients and healthcare providers and far greater convenience for patients. Additionally, the simplicity of using a single drug/device protocol to successfully address multiple aspects of COVID-19 intervention, reduced the inherent risks of drug interactions and reduced the costs and other associated challenges of a poly-drug regimen. Despite the markedly high viral loads and morbidity at presentation, in-patient hospital admission was avoided in all pilot subjects who completed the protocol. Reducing the need for hospital admission represents a significant benefit to patients while reducing the burden on local health care facilities.

Based on the above factors and the broad scope of PDI application, our protocol can potentially be expanded to target not only an unlimited number of future COVID-19 variants, but also many other infections contracted via the upper respiratory tract or oral mucosa in the future. While the data presented are indeed promising, further extensive studies with larger cohorts utilizing our standardized LURP are warranted to validate our results.

## 6. Patents

An international patent was filed resulting from the work reported in this manuscript: International Patent Application No. PCT/EP2021/062061-SCHIKORA, Detlef; Luminnova Health.

## Figures and Tables

**Table 1:** Mechanisms of action of methylene blue (MB) in COVID-19 disease. Abbreviations: ACE2: Angiotensin-converting enzyme 2.

Actions against SARS-CoV-2 Virus	
Reduced Viral Entry	Inhibits binding of spike protein to ACE2 receptor Impairs membrane fusion/endocytosis
Reduced Viral Replication	Reduces viral uncoating (increases lysosomal pH) Reduces protein translation (increases lysosomal pH) Inhibits RNA dependent RNA polymerase (zinc ionophore)
Photo-Oxidative Viral Inactivation [activation by 660 nm light]	Targets viraemia Augments the effects of topical oral/nasal PDI
Reduced Organ Damage	
Reduced Cytopathic Effects	Reduced viral replication/protein translation Reduced oxidative stress Reduced cytokine damage
Reduced Hypoxia	Improves mitochondrial respiration Rapidly reduces methemoglobinemia Reduces micro-thrombi (reduces platelet aggregation)
Reduced Hyper-Inflammation	Inhibits NLRP3 Reduces excess nitric oxide/bradykinin activity Scavenger of reactive oxygen/nitrogen species
Broad Spectrum Antimicrobial (Bacterial/Fungal/Other Viruses)	
Reduced Secondary Infections	Intrinsic anti-microbial actions Intravascular photo-oxidative anti-microbial actions

**Table 2:** Shows the device description and LED dosimetry and treatment protocols for photodynamic inactivation (PDI).

Type of Device	Semi-Circular Non-Thermal LED Device
Light source	Light Emitting Diodes
Beam type	Non-Coherent
Wavelength	660 nm
Operation emission mode	Continuous mode
Number of LEDs	24
Irradiance per each LED	5.5m W/cm^2^
Spot size per each LED	0.045 cm^2^
Power output per each LED	122 mW
Total power output	2928 mW
Aperture (surface area of the device)	18 cm^2^
Total power density (irradiance)	163 mW/cm^2^
Treatment Time	5 min (300 s)
Energy density	49 J/cm^2^
Treatment interval	Variable: between 8–24 h (hours)

**Table 3:** Shows the device description and LED dosimetry and treatment protocols for sublingual photobiomodulation therapy.

Type of Device	Semi-Circular Non-Thermal LED Device
Light source	Light Emitting Diodes
Beam type	Non-Coherent
Wavelength	660 nm
Operation emission mode	Continuous mode
Number of LEDs	6
Irradiance per each LED	5.5 mW/cm^2^
Spot size per LED	0.045 cm^2^
Power output per each LED	122 mW
Total power output	732 mW
Device aperture (over sublingual vein)	9 cm^2^
Total power density (irradiance)	81 mW/cm^2^
Total treatment duration	5 min (300 s)
Estimated blood exposure	60 s
Energy density (blood)	4.9 J/cm^2^
Treatment interval	Variable: between 8–24 h (hours)

**Table 4:** Evolution of COVID-19 Treatment Protocol from August 2020 to July 2021.

Intervention	August 2020–March 2021	June/July 2021 (Pilot Study)	LUMINNOVA Upper Respiratory Protocol
Oral MB	Optional: used if symptoms were severe/delayed presentation	Initially 1 mg/kg administered 12-hourly.	Up to 2 mg/kg administered 8-hourly for a total of 8 treatments (unless contraindicated or very mild presentation).
Sublingual PBM	Not utilized	Initially utilized for a patient that had not adequately responded to oral MB and in a case where oral MB was contraindicated.	Administered eight treatments every 8 h or until symptoms were resolved
Oral/nasal PDT	Total of two treatment sessions 12 h apart.	The number of treatment sessions gradually evolved as we observed the changes in CT values each day.	Treatment course consisted of eight sessions carried out every 8 h.

**Table 5:** Shows the summary of one-year follow-up responses. * COVID-19 re-infection after recovery from initial infection. ** Repeat positive SARS-CoV-2 test after recovery from initial infection. Abbreviations: PASC: post-acute sequelae of COVID-19.

Subject	Post-Acute Sequelae of SARS-CoV-2	Symptomatic COVID-19 Re-Infection *	Repeat Positive SARS-CoV-2 Test **
1	No PASC	No	No
2	No PASC	No	No
3	No PASC	No	No
4	No PASC	No	No
5	Significant PASC symptoms	Yes	Yes
6	No PASC	No	No
7	No PASC	No	No
8	Mild PASC symptoms	No	No

**Table 6:** Summary of the pilot study data and results. * Based on the difference in SARS gene initial CT value and 34 (threshold for a negative test result). Abbreviations: CT: cycle threshold; M: male; F: female; yrs: years; no.: number; PCR: polymerase chain reaction; BMI: body mass index.

Case No. (Subject)	Date	Age (yrs)	M/F	Co-Morbidity	Days after Onset	Initial CT Value	Viral Load Reduction Factor *	Days to Negative PCR
1	26 June 2021	61	F	BMI 31.1 Recurrent Bronchitis	8	16.34/17.19	207,000	7
2	6 July 2021	55	M	BMI 34 Hypertension Smoker	5	20.17/22.12	-	Clinically recovered within 12 h (Refused repeat test)
3	6 July 2021	40	M	BMI 31.7	5	18.99/20.43	32,768	4
4	10 July 2021	38	F	BMI 31.3	3	11.96/15.97	4,194,304	4
5	17 July 2021	42	M	BMI 32.5	5	18.14/19.24	-	Did not complete treatment protocol. Hospitalized 3 days after discontinuing treatment
6	18 July 2021	31	F	BMI 27.6 Pregnant 8 months gestation	5	15.21/16.62	450,000	7
7	21 July 2021	47	F	BMI 29.2	2	26.96/29.04	128	4
8	28 July 2021	38	M	BMI 32.5	3	6.05/9.41	259,000,000	8 (Follow up testing was delayed)

## Data Availability

All the Data Availability Statements are included in the paper.
